# Metastatic Lymph Node Ratio for Predicting Recurrence in Medullary Thyroid Cancer

**DOI:** 10.3390/cancers13225842

**Published:** 2021-11-21

**Authors:** Jinyoung Kim, Jun Park, Hyunju Park, Min Sun Choi, Hye Won Jang, Tae Hyuk Kim, Sun Wook Kim, Jae Hoon Chung

**Affiliations:** 1 Division of Endocrinology and Metabolism, Department of Medicine, Thyroid Center, Samsung Medical Center, Sungkyunkwan University School of Medicine, Seoul 06351, Korea; j513kim.md@gmail.com (J.K.); pjun113@gmail.com (J.P.); hj1006.park@samsung.com (H.P.); msun1919@naver.com (M.S.C.); taehyukmd.kim@samsung.com (T.H.K.); sunwooksmc.kim@samsung.com (S.W.K.); 2 Department of Medical Education, Sungkyunkwan University School of Medicine, Seoul 06351, Korea; jhw463@skku.edu

**Keywords:** thyroid neoplasms, prognosis, lymph node ratio

## Abstract

**Simple Summary:**

The anatomical staging system for thyroid cancer only contains categories for lymph node compartments. The metastatic lymph node ratio (LNR), which is the ratio of metastasized lymph nodes to the total number of evaluated lymph nodes, is suggested as a quantitative evaluation tool for lymph node metastasis in patients with medullary thyroid cancer in this study. The initial stratification implemented in this study was helpful in predicting structural recurrence, and LNR was identified as a predictor of disease-free survival.

**Abstract:**

The lymph node ratio (LNR) has been investigated as a prognostic factor in many different types of cancers, including differentiated thyroid cancer; however, reports regarding medullary thyroid cancer (MTC) are limited. Therefore, this study aims to evaluate LNR as a risk factor for structural recurrence in patients with MTC. Medical records of patients treated for MTC in a single tertiary center between 1995 and 2017 were retrospectively reviewed. LNR is defined as the number of metastatic lymph nodes or lymph node metastases (LNM) divided by the number of retrieved lymph nodes or lymph node yield (LNY). In the survival analysis, recurrence-free survival was defined as the time from the date of total thyroidectomy to recurrence or last follow-up. To identify risk factors influencing structural recurrence, univariable and multivariable Cox proportional hazard models were used. A total of 132 patients were enrolled. The mean age of study participants was 49.7 years, and 86 patients (65%) were women. Structural recurrence was identified in 39 patients at the end of the study period, and the median follow-up period was 8.7 years. In univariable analyses, gross extra thyroidal extension, N stage, postoperative serum calcitonin and carcinoembryonic antigen (CEA) levels, and LNR were significant (*p* < 0.05) predictors of structural recurrence. In multivariable analysis, postoperative serum calcitonin, postoperative serum CEA, and LNR were identified as a predictor of disease-free survival (*p* < 0.05). LNR can potentially predict structural recurrence as a quantitative evaluation tool for lymph node metastasis in patients with MTC.

## 1. Introduction

Medullary thyroid cancer (MTC) consists of thyroid parafollicular cells and has distinct biological features [1]. Unlike the effects observed in differentiated thyroid cancer (DTC), thyroid-stimulating hormone suppression is not effective for MTC treatment because parafollicular cells do not have the thyroid-stimulating hormone receptor [2]. Furthermore, the response to radioactive iodine therapy as an adjuvant treatment after total thyroidectomy is poor [3]. Therefore, postoperative evaluation for initial risk stratification is crucial for predicting prognosis in patients with MTC [4].

According to the American Joint Committee on Cancer (AJCC), the lymph node stages in patients with MTC are classified as follows: pN0, no evidence of locoregional lymph node metastasis; pN1a, metastasis to level VI or VII lymph nodes; and pN1b, metastasis to the unilateral, bilateral, or contralateral neck or retropharyngeal lymph nodes [5]. The AJCC staging system only contains categories for lymph node compartments; therefore, previous studies have attempted to quantitatively assess lymph node status [6,7,8,9]. In some of them, increased positive lymph node ratio (LNR) in the postoperative evaluation was suggested to be associated with poor prognosis [10,11,12].

A staging system that groups patients according to the absolute number of lymph nodes could improve the risk stratification for recurrence [7,8]. However, this process could be limited by case variance, pathological identification, and surgical techniques [13,14]. Variation in these factors may result in a wide range of the total number of metastatic lymph nodes, thereby influencing staging. The LNR, which is the ratio of metastasized lymph nodes to the total number of evaluated lymph nodes, has been suggested to avoid this discrepancy.

The LNR is a prognostic predictor in many other cancers, including DTC [15,16,17,18,19]; however, reports for MTC are rare. Therefore, this study aims to evaluate LNR as a risk factor for structural recurrence in patients with MTC.

## 2. Materials and Methods

**Figure 1:**
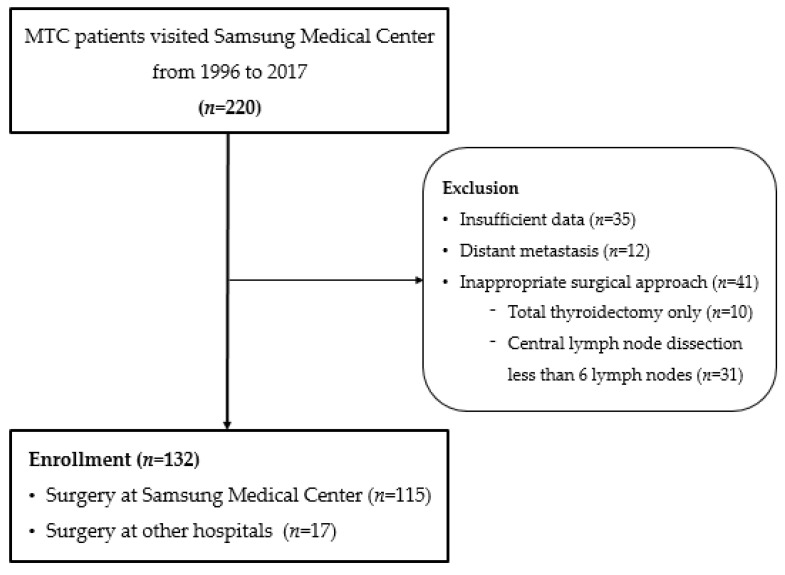
Flow chart showing sample selection.

The medical records of patients treated for MTC at a single tertiary center between 1995 and 2017 were reviewed. The study was conducted after obtaining approval from the Institutional Review Board (SMC 2020-07-135), and informed consent was exempted due to its retrospective study design. Patients who underwent total thyroidectomy with neck dissection for treatment were included, and patients with distant metastasis were excluded (Figure 1).

Following the American Thyroid Association guidelines [2], all patients underwent total thyroidectomy and central compartment dissection (level VI) even when metastatic lymph nodes in the neck were not detected on preoperative imaging. In cases where metastatic lymph nodes in the lateral neck were identified before surgery, modified radical neck dissection was performed. Even if only ipsilateral involvement was confirmed, contralateral dissection was considered for patients with a preoperative calcitonin level of >200 pg/mL. Neck dissection with less than six lymph nodes in pathological confirmations following surgery for a specific reason, such as uncertain diagnosis and tumor locations, was considered as insufficient surgery [20] and excluded (Figure 1) The extent of surgery was classified according to the type of neck dissection (central, ipsilateral, or bilateral) performed with total thyroidectomy.

LNR was defined as the number of metastatic lymph nodes (LNMs) divided by to the number of retrieved lymph nodes (lymph node yield, LNY). Serum calcitonin was measured using immunoradiometric assay (CT-US-IRMA, DIAsource ImmunoAssays S.A., Louvain-la-Neuve, Belgium). Considering the functional sensitivity of the test, serum calcitonin of <5.0 pg/mL 3 months following total thyroidectomy was defined as normal. Serum carcinoembryonic antigen (CEA) was measured by radiometric assay using a commercial assay kit (CEA-RAICT, Cisbio biointernational, Gif-sur-Yvette, France). For serum CEA, 5 ng/mL was used as the cut-off of normal range following surgery. Local recurrence was diagnosed using sonography and fine-needle aspiration.

Continuous variables that satisfied a normal distribution are described as mean and standard deviation (SD), and continuous variables that did not follow a normal distribution are presented as the median and interquartile range (IQR). Categorical variables are expressed as numbers and percentages. In survival analysis, recurrence-free survival was defined as the time from the date of total thyroidectomy to structural recurrence or last follow-up. Structural recurrence was defined as metastatic lymph nodes confirmed by re-operation or distant metastasis confirmed by computed tomography (CT) or positron emission tomography (PET). To identify risk factors influencing structural recurrence, univariable and multivariable Cox proportional hazard models were applied. The concordance index (C-index) was calculated to compare prediction performance [21]. R version 4.0.2. (R Foundation for Statistical Computing, Vienna, Austria) was used for statistical analysis.

## 3. Results

### 3.1. Baseline Characteristics of the Study Population

A total of 132 patients were enrolled, with a mean age of 49.7 years, and 86 patients (65%) were women. Eighteen patients (13.7%) were the hereditary type, with a confirmed family history. The mean size of the largest tumor diameter was 1.98 cm, and 25 patients (19%) had a gross extra thyroidal extension. Twenty-two patients (17%) had bilateral tumors. When the lymph node status was evaluated, central LNM was identified in 23 patients (17.4%) and lateral LNM in 59 patients (44.7%). Structural recurrence was identified in 39 patients at the end of the study period, and the median follow-up period was 8.7 years (Table 1).

### 3.2. Differences between the Number and Ratio of Metastatic Lymph Nodes (LNM vs. LNR)

**Figure 2:**
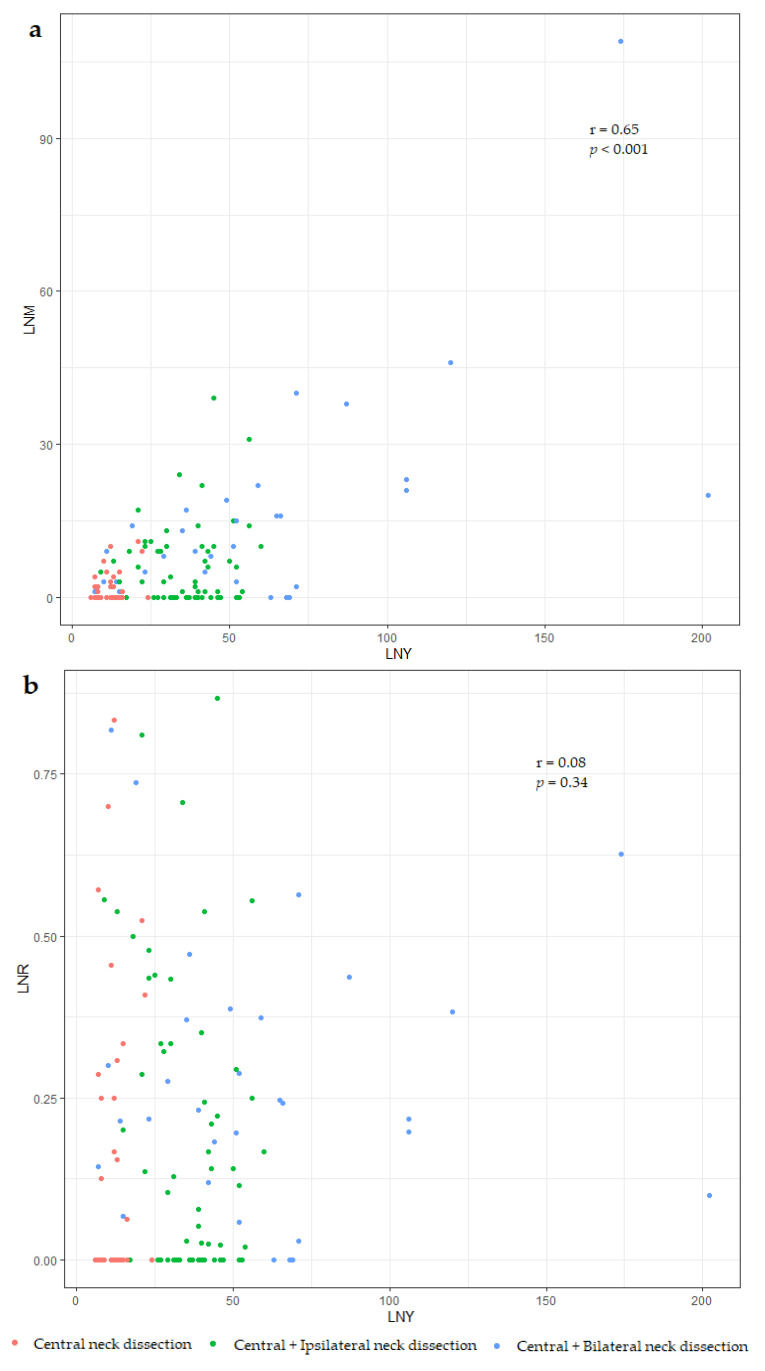
Relationship between lymph node metastases (LNMs) and the number of lymph nodes resected or lymph node yield (LNY). LNM and LNY were significantly related (**a**). Meanwhile, the relationship between lymph node ratio (LNR) and lymph node yield (LNY) was distributed independently (**b**).

LNM increases were significantly associated with increased LNY; thus, as LNY increases, LNM also tends to increase along with surgical extent (r = 0.65, *p* < 0.001). Meanwhile, LNR did not show any association with LNY, indicating that it could be a factor not affected by surgery (r = 0.08, *p* = 0.34) (Figure 2).

Univariable Cox analysis to predict structural recurrence after the initial surgery was performed using N stage, LNM, and LNR. The C-index values were calculated as 0.704, 0.760, and 0.799, respectively, with the C-index for LNR being the highest among the three models. The LNM and LNR variables were continuous, and they had significantly higher C-index values than the model using the categorical variable (N stage) (Table 2). By performing subgroup analysis according to N stages, LNM was found to be only significant at the N1b stage. LNR was a significant factor in both N1a and N1b (Table 3).

### 3.3. Prognostic Factors Influencing Disease-Free Survival

Survival analysis was performed to identify the clinical characteristics affecting structural recurrence using univariable and multivariable Cox regression models. In univariable analyses, gross extrathyroidal extension, N stage, LNR, and postoperative serum calcitonin level were significant (*p* < 0.05) factors. When the multivariable analysis was performed, postoperative serum calcitonin, postoperative serum CEA, and LNR were identified as predictors of disease-free survival (*p* < 0.05) (Table 4).

The C-index of Model 1, including only known anatomical variables, was 0.762. Considering the impact of initial surgery, postoperative serum calcitonin level and LNR were consecutively added to Model 1. As each variable was added, C-index values increased to 0.797 and 0.810; however, the extent of increase was not statistically significant (*p* = 0.100 and *p* = 0.089). The C-index of Model 2, including all significant variables added to Model 1, yielded a maximum C-index of 0.850, which was statistically significant (*p* = 0.002) (Table 5).

### 3.4. Determining the Cut-Off Value of LNR

To estimate the cut-off point for classifying high-risk patients in clinical situations, univariable Cox analysis was performed by changing the reference point at 0.05 intervals. By comparing these C-indices, an LNR of 0.20 was determined as the cut-off level at the level with the highest predictive performance (C-index = 0.750) and was thus considered the optimal cut-off in this study cohort (Table 6).

LNR increased in proportion to the N stage and was confirmed to have high levels in advanced MTC, with large size and high lymph node stages through additional analysis of patients at high risk for LNR recurrence (Table 7).

**Figure 3:**
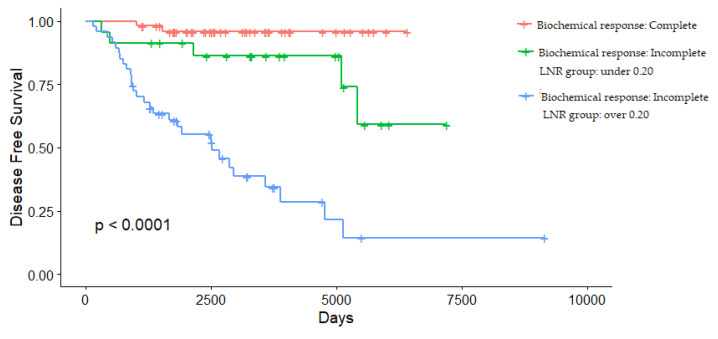
Disease-free survival stratified by biochemical response and LNR group.

The LNR can further stratify recurrence risk in patients with a biochemical incomplete response (Figure 3).

## 4. Discussion

Extensive surgery involving total thyroidectomy with central lymph node dissection is recommended as the initial treatment for patients with MTC as the condition is refractory to most medical therapies [3,22]. In the evaluation of recurrence following surgery, tumor-node-metastasis staging is an established prognostic approach [23]. However, traditional anatomical staging has a drawback as it cannot reflect the effects of surgery as an initial treatment and the quantitative status of metastatic lymph nodes. This study suggests the clinical value of metastatic LNR as a prognostic factor in the initial stratification of MTC.

LNM can also be considered for quantitative lymph node evaluation to present a consistent system since the absolute number of metastatic lymph nodes is presented in the initial risk stratification of DTC [7,24]. However, it was thought to have limited predictive power as it is affected by the initial surgery and tends to increase in proportion to LNY (Figure 2) [25]. LNR is calculated by dividing the number of metastatic lymph nodes by the total number of examined lymph nodes, regardless of the quality of neck dissection and pathologic examination. Therefore, LNR was proposed as a method to correct the effects of surgery. LNM was insignificant at the N1a stage in a subgroup analysis of this study population, whereas LNR was a significant prognostic factor in both N1a and N1b (Table 3). As patients who underwent insufficient dissection or with distant metastasis were excluded in this study, LNR can provide much more significant predictive power in actual clinical situations. Log-odds of LNR (LODDS) values and simple LNRs were suggested as prognostic factors in various types of cancers [26,27]. LODDS was also used, not N stage or the number of pathologically positive lymph nodes, in the nomogram for the risk assessment of recurrence in a previous study on MTC [9]. However, LNR is thought to be easier for intuitive understanding in clinical situations because LODDS entails additional mathematical calculations [28]. As LNR provided sufficient statistical predictive power in the analysis results, further analysis of LODSS was not addressed in this study.

Leggett et al. presented the first study on the prognostic LNR value in MTC [10]. LNY was also analyzed as an important prognostic factor in clinically N-positive patients. This was emphasized as a basis for a wide range of operations for surgeons, combined with the suggestion that systematic compartment-oriented dissection is required rather than berry-picking-type positive lymph node resection in MTC [29]. However, extensive neck dissection may increase the risk of complications [30]. In addition, they suggested that increased LNY was not associated with improved survival and that patient’s age or tumor size could be more significant for the patient’s survival. We believe that surgeons who treated patients with MTC did their best with the instructed guidelines, and patients with insufficient surgery were also excluded because of uncertain diagnosis or tumor locations. Therefore, we considered LNR as one of the characteristics of diseases, such as the N stage in this study, and LNR significantly predicted disease-free survival, regardless of patient’s age or tumor size (Table 5). When considering disease-specific mortality, LNR ineffectively predicted patient survival in this study cohort. Therefore, extensive lymph node dissection is thought to reduce recurrence, but it can be controversial when considering patient survival and quality of life.

The prognosis worsens as LNR increases; however, we further investigated the cut-off for easier clinical application. In this study cohort, the LNR cut-off value was 0.20 to differentiate the high- and low-risk groups for structural recurrence with the highest C-index (Table 6). However, several previous studies have proposed different LNR cut-off values. A previous study suggested a cut-off of 0.10 [11]. Another study suggested 0.50 as the cut-off for disease-specific survival [12]. In DTC, cut-offs of 0.18 [18] and 0.42 [31] were suggested in various studies. Therefore, LNR cut-offs for recurrence and survival should be clarified by conducting a study with a longer follow-up period in a large population. Meanwhile, a study suggesting LNR as a prognostic factor reported that MTC is a more significant prognostic factor in elderly patients with sporadic MTC [11]. However, LNR did not significantly correlate with age or hereditary status in the analysis of this study. (Table 7) Therefore, we propose LNR as a characteristic only associated with disease burden.

Serum calcitonin and CEA monitoring are recommended for postoperative surveillance [2], and postoperative serum calcitonin has been suggested as a significant prognostic factor in previous studies [32,33,34]. Postoperative serum calcitonin and CEA were included in the analysis of this study as the well-known biomarkers for MTC, and the postoperative serum calcitonin and CEA level provided meaningful information in predicting recurrence in this study (Table 5). Some previous studies have evaluated the association between the degree of LNM and serum calcitonin normalization [35,36]. In a group of N1b-stage patients of this study cohort, serum calcitonin was not normalized in 50 of 59 patients (84.7%). The LNR may help to further stratify recurrence risk, especially in patients with N1b-stage disease (Figure 3).

The initial stratification implemented in this study was helpful in predicting structural recurrence. However, it is necessary to note that two of the patients who showed recurrence in this study nevertheless had excellent biochemical responses and low LNRs, although postoperative serum calcitonin levels and LNR offer effective predictive power for structural recurrence. Repetitive follow-up has been suggested to be important for patients with MTC, even for low-risk patients with MTC. For surveillance of exceptional cases during initial risk stratification and those with unpredictable rapid progression, serial measurement of biomarkers, such as serum calcitonin and CEA, and calculation of the doubling time are recommended [37].

The limitation of this study is its retrospective design in a single center. As only patients with sufficient clinical information were analyzed, the possibility of selection bias cannot be denied. However, considering the prevalence of MTC, a relatively large number of study populations were included and a median of 8.7 (range: 3–26) years is a sufficient period to observe recurrence.

## 5. Conclusions

LNR can potentially predict structural recurrence as a quantitative evaluation tool for LNM in patients with MTC.

## Figures and Tables

**Table 1:** Baseline characteristics of the study cohort.

Number	132
Age, years (mean ± SD) Sex, women (%)	49.7 ± 13.9 86 (65)
Hereditary type, number (%)	18 (13.7)
Size, cm (mean ± SD) Gross extra thyroidal extension, number (%)	1.98 ± 1.39 25 (19)
Bilateral tumors, number (%)	22 (17)
T stage, number (%)	
1a	35 (26.5)
1b	35 (26.5)
2	27 (20.5)
3a	7 (5.3)
3b	17 (12.9)
4a	11 (8.3)
N stage, number (%)	
0	50 (37.9)
1a	23 (17.4)
1b	59 (44.7)
AJCC 8th stage, number (%)	
I	37 (28.0)
II	13 (9.8)
III	23 (17.4)
IVA	59 (44.7)
The surgical extent of neck dissection, number (%) Central neck dissection Central + Ipsilateral neck dissection Central + Bilateral neck dissection	37 (28.0) 69 (52.3) 26 (19.7)
Lymph node status Metastasized lymph nodes, number (median (IQR)) Resected lymph nodes, number (median (IQR)) Lymph node ratio, number (median (IQR))	2.5 (0–10) 29 (13–44) 0.122 (0–0.311)
Follow-up duration, years (median (IQR))	8.7 (5.5–12.9)

SD, standard deviation; AJCC, American Joint Committee on Cancer; IQR, interquartile range.

**Table 2:** Comparison of N stage, LNM, and LNR.

	N Stage	LNM	LNR
C-index (SD)	0.704 (0.034)	0.760 (0.039)	0.799 (0.036)
*p*	(reference)	0.023	0.002

SD, standard deviation; LNM, the number of metastatic lymph nodes; LNR, metastatic lymph node ratio.

**Table 3:** Comparison of LNM and LNR based on N stage.

	N Stage	Median (IQR)	HR (95% CI)	*p*
LNM	N1a	2 (1–4)	1.28 (0.99–1.64)	0.056
(number)	N1b	10 (6.5–16.5)	1.02 (1.01–1.04)	0.001
LNR	N1a	15.4 (4.6–38.2)	1.04 (1.01–1.08)	0.010
(%)	N1b	28.8 (20.0–43.6)	1.05 (1.03–1.07)	<0.001

IQR, interquartile range; HR, hazard ratio; CI, confidence interval; LNM, the number of metastatic lymph nodes; LNR, metastatic lymph node ratio.

**Table 4:** Univariate and multivariate Cox regression models for predicting structural recurrence.

Univariate Analysis			Multivariate Analysis (Model 1)		Multivariate Analysis (Model 2)	
	HR (95% CI)	*p*	HR (95% CI)	*p*	HR (95% CI)	*p*
Age	0.98 (0.95–1.00)	0.057				
Sex, men	1.39 (0.73–2.64)	0.314				
Size	1.19 (0.99–1.42)	0.062				
Gross ETE, positive	5.17 (2.74–9.77)	<0.001	2.89 (1.47–5.69)	0.002	1.56 (0.75–3.21)	0.232
N stage 0 1a 1b	1.00 (reference) 12.96 (2.78–60.4) 15.42 (3.66–65.2)	0.001 <0.001	8.94 (1.79–44.8) 11.0 (2.52–48.1)	0.008 0.001	4.76 (0.91–24.7) 1.16 (0.20–6.75)	0.064 0.868
LNR (%)	1.05 (1.04–1.06)	<0.001			1.04 (1.02–1.06)	<0.001
Postoperative calcitonin ≥ 5 pg/mL	15.76 (3.80–65.40)	<0.001			5.43 (1.13–26.1)	0.035
Postoperative CEA ≥5 ng/mL	7.79 (4.11–14.77)	<0.001			2.83 (1.39–5.78)	0.004

HR, hazard ratio; CI, confidence interval; ETE, extra thyroidal extension; LNR, metastatic lymph node ratio; CEA, carcinoembryonic antigen.

**Table 5:** Comparison of predictive power between models.

	Model 1	Model 1 +Postoperative Calcitonin	Model 1 +Lymph Node Ratio	Model 2
C-index (SD)	0.762 (0.036)	0.797 (0.034)	0.810 (0.036)	0.850 (0.034)
*p*	(reference)	0.100	0.089	0.002

SD, standard deviation.

**Table 6:** Univariate Cox regression analysis for determining the cut-off value of LNR.

LNR Cut-Off	HR	95% CI	*p*	C-Index (Standard Error)
0.05	16.89	4.05–70.38	<0.001	0.696 (0.028)
0.10	22.45	5.38–93.64	<0.001	0.728 (0.027)
0.15	11.57	4.50–29.74	<0.001	0.741 (0.031)
0.20	10.29	4.50–23.53	<0.001	0.750 (0.034)
0.25	9.07	4.36–18.87	<0.001	0.733 (0.039)
0.30	5.71	2.99–10.91	<0.001	0.689 (0.040)
0.35	5.82	3.09–10.96	<0.001	0.687 (0.040)
0.40	6.12	3.22–11.62	<0.001	0.668 (0.040)
0.45	5.08	2.64–9.77	<0.001	0.629 (0.038)
0.50	5.44	2.81–10.54	<0.001	0.623 (0.037)

LNR, metastatic lymph node ratio; HR, hazard ratio; CI, confidence interval.

**Table 7:** Baseline characteristics according to lymph node ratio group.

Variables	LNR < 0.20 (n = 79)	LNR ≥ 0.20 (n = 53)	*p*
Age (mean ± SD)	50.67 (12.70)	48.30 (15.60)	0.340
Sex, men (%)	23 (29.1)	23 (43.4)	0.133
Size, cm (mean ± SD)	1.72 (1.08)	2.38 (1.68)	<0.001
Gross extrathyroidal extension, number (%)	4 (5.1)	21 (39.6)	0.007
Bilaterality, number (%)	13 (16.5)	9 (17.0)	1.000
N stage			<0.001
0	49 (62.0)	0 (0.00	
1a	14 (17.7)	9 (17.0)	
1b	16 (20.3)	44 (83.0)	
Postoperative calcitonin ≥ 5pg/mL	23 (29.1)	51 (96.2)	<0.001

SD, standard deviation; LNR, metastatic lymph node ratio.

## Data Availability

The data presented in this study are available upon reasonable request from the corresponding author.
